# Low Helicobacter pylori primary resistance to clarithromycin in gastric biopsy specimens from dyspeptic patients of a city in the interior of São Paulo, Brazil

**DOI:** 10.1186/1471-230X-13-164

**Published:** 2013-12-04

**Authors:** Rodrigo Buzinaro Suzuki, Rodrigo Augusto Basso Lopes, George Arouche da Câmara Lopes, Tin Hung Ho, Márcia Aparecida Sperança

**Affiliations:** 1 Department of Molecular Biology, Marilia Medical School, Marilia, São Paulo, Brazil; 2 Center of Natural and Human Sciences, Universidade Federal do ABC, Santo André, São Paulo, Brazil

**Keywords:** *Helicobacter pylori*, Clarithromycin resistance, *Helicobacter pylori* 23S rDNA, Gastric diseases, Nucleic acid based diagnostic

## Abstract

**Background:**

Clarithromycin, amoxicillin, and a pump proton inhibitor are the most common drugs recommended as first-line triple therapy for *H.pylori* treatment, which results in eradication rates close to 80%, varying regionally, principally due to emergency cases and increases of clarithromycin resistant strains. Nucleotide substitutions at the *H. pylori* domain V of the 23S rRNA fraction are involved in the macrolide resistance and the A2142G and A2143G mutations are predominant in clinical isolates worldwide including in Brazil. As *H. pylori* culture is fastidious, we investigated the primary occurrence of *H. pylori* A2142G and A2143G rDNA 23S mutations using a molecular approach directly on gastric biopsies of dyspeptic patients consecutively attended at Hospital das Clinicas of Marilia, São Paulo, Brazil.

**Methods:**

Biopsy specimens obtained from 1137 dyspeptic patients, were subjected to histopathology and *H. pylori* diagnosis by histology and PCR. PCR/RFLP assay was used to detect A2142G and A2143G point mutations at domain V of the *H. pylori* 23S rDNA associated with clarithromycin resistance. Through the developed assay, a 768 bp PCR amplicon corresponding to1728 to 2495 bp of the 23S *H. pylori* rDNA is restricted with M*boII* for A2142G mutation detection and with B*saI* for A2143G mutation detection. Occurrence of 23S rDNA A2142G results in two DNA fragments (418 and 350 bp) and of 23S rDNA A2143G results in three DNA fragments (108, 310 and 350pb), due to a conserved B*saI* restriction site.

**Results:**

The PCR method used to diagnose *H. pylori* presented sensitivity, specificity and accuracy of 77,6%, 79,3% and 78,6%, respectively, compared to histology, the gold standard method for *H. pylori* diagnosis used in our routine. Prevalence of *H.pylori* with clarithromycin resistant genotypes was 2,46%, with predominance of A2143G 23S rDNA point mutation.

**Conclusions:**

The PCR/RFLP assay was a rapid and accurate *H.pylori* diagnostic and clarithromycin resistance determination method useful for routine practice. As prevalence of primary resistance of *H.pylori* to clarithromycin due to A2142G and A2143G mutations remains low in Marilia, the standard clarithromycin containing triple therapy is still valid.

## Background

It is widely accepted that *Helicobacter pylori*, a Gram negative microaerophylic bacterium, is involved in several clinical digestive tract conditions such as chronic gastritis, peptic and duodenal ulcers, gastric cancer and lymphoproliferative disorders [1]. Treatment of *H. pylori* infection results in ulcer healing and in a reduction of the risk of gastric cancer and lymphoma [2,3].

Once the bacterium *H. pylori* is detected in altered gastric mucosa, the indicated treatment consists of a triple antibiotic regimen including methronidazol, clarithromycin, amoxicillin, tinidazole, tetracycline and fluoroquinolones associated with a pump proton inhibitor such as omeprazol, lansoprazol or pantoprazol [4-6]. *H. pylori* eradication rates with a number of combined agents and regimens are close to 80% [7-9], varying from country to country and regionally, within countries [10]. Several factors contribute to this low rate of *H. pylori* healing including the inefficiency of the antibiotic penetration in the gastric mucosa, inactivation of the antibiotic by the acid secretion of the stomach [11], lack of the patient compliance [12] and principally, emergency cases and increasing *H. pylori* antibiotic resistant strains [13]. Thus, regional *H. pylori* resistance surveillance is of great importance for test and treatment strategies.

In Brazil, a country of continental dimensions, the majority of practicing clinicians employ the classical triple regimen composed of clarithromycin, amoxicillin and a proton pump inhibitor for seven days as first line therapy to overcome *H. pylori* infection [5,14]. This regimen has been proved to become inefficient worldwide, mainly as a result of the emergence and increase of *H. pylori* strains resistant to clarithromycin, which reduces the bacterium treatment efficiency from 55% to 100% [15-18]. Among Brazilian localities, *H. pylori* clarithromycin resistance presents high prevalence, varying from 7-16% in adults [19-22] and 27% in children [23]. Accordingly, considering the clinical importance of primary *H. pylori* resistance to clarithromycin, its prevalence should be considered before choosing eradication regimens [24].

Determination of *H. pylory in vitro* susceptibility to antibiotics can be performed by standard techniques such as the agar diffusion, agar dilution and broth microdilution methods and the E-test. However, because of the slow growth and the particular requirements of *H.pylori* culture, this approach is not reliable for use in most routine clinical laboratories, principally in developing countries. Hence, molecular tests targeting *H. pylori* resistance associated gene mutations directly from gastric biopsy specimens have the potential for use in large scale studies [25-29].

The molecular mechanism involved in clarithromycin resistance consists of mutations in the sequence of the *H. pylori* domain V of the 23S rRNA fraction which is involved in the peptiltransferase ribosome binding site preventing the ligation of the macrolide to the rRNA [30]. The major characterized point mutations are A to G at positions 2142 and 2143, A to C at 2142 [31-33], A to T at 2144 [34], T to C at 2717 [35] and C to A at 2694 [36]. The A2142G and A2143G mutations are predominant in clinical isolates worldwide including in Brazil [21,37-40]. Thus, in order to perform a large scale investigation of clarithromycin primary resistance directly from biopsy specimens of 1137 patients attended at the Hospital das Clínicas of Marilia, a city in the interior of São Paulo, Brazil, we developed a polymerase chain reaction associated with restriction fragment length polymorphism (PCR-RFLP) assay to detect the A2142G and A2143G nucleotide substitutions at domain V of the *H. pylori* 23S rDNA.

## Methods

### Patients

1137 adult patients resident in Marilia city, São Paulo State of Brazil, aged 19 to 91 years, who had consecutively undergone esophagogastroduodenoscopy (EGD) for upper abdominal pain or dyspeptic symptoms from February 2003 through December 2006 at the gastroenterology outpatient clinic of the Hospital das Clínicas of Marília Medical School, were enrolled in this study.

### Endoscopy and biopsies

The EGD was accomplished by fibroendoscope (GIF-XP20, GIF-XQ20) or video-endoscope (GIF-100) both from Olympus, Shinjuku-ku, Tokyo, Japan. Gastric or duodenal ulcer diagnostic was defined by endoscopy and two fragments of the antrum were collected to perform the rapid urease and histopathological tests. The biopsy used for the rapid urease test was further submitted to DNA extraction. The protocol used is in agreement with the Helsinki Declaration and was approved by the Ethical Committee in Human Research from Marilia Medical School, under reference number 388/01. In the Ethical Committee approved research protocol a written informed consent from each patient included in this study was waived as all gastric biopsy samples analyzed were the same biopsies used routinely for urease rapid test as part of the gastroenterology outpatient service of the Hospital das Clinicas of Marilia Medical School and thus, no specific patient intervention was necessary for the enrollment in this proposed study. Accordingly, waiver of the written informed consent did not adversely affected the rights and welfare of the subjects included in this research, and also the confidentiality of the patients identity was guaranteed.

### Histology

One antral specimen was fixed in formol solution at 10% and embedded in paraffin. Sections were Giemsa stained for *H. pylori* evaluation and were stained with hematoxilin and eosin for assessment of histopathologic alterations [41].

### Polymerase chain reaction, restriction and sequencing analysis

**Figure 1:**
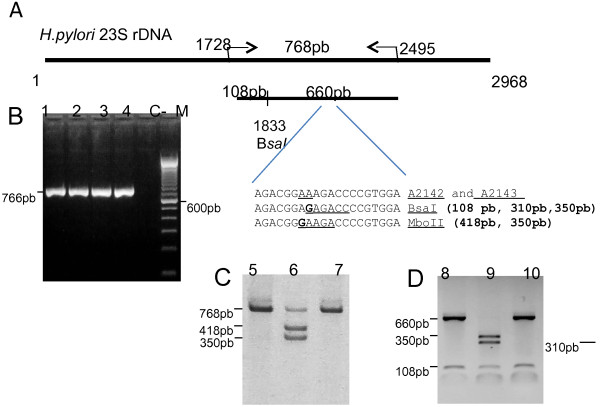
**Molecular diagnosis of *Helicobacter pylori *by PCR and RLFP detection of the domain V of the 23S rRNA mutations A2142G and A2143G responsible for clarithromycin resistance. A.** Representation of the *H. pylori* 23S coding gene (Genbank:HPU27270), position of the primers used for PCR and of the A2142 and A2143 from fraction V of the 23S rDNA, size of the fragments obtained with restriction enzymes BsaI and M*boII* in PCR fragments containing the mutations A2142G and A2143G, and internal B*saI* restriction site of the 768 bp amplicon, are indicated. **B, C** and **D.** Agarose gel stained with ethidium bromide containing a PCR diagnostic analysis, restriction analysis of the 768pb amplicon with M*boII* and B*saI*, respectively. 1–10 correspond to different human biopsy specimens; C-, negative control of PCR, M-100 bp ladder purchased from Invitrogen. Sizes of the DNA fragments are indicated on the left or right of each gel figure.

The same biopsy used for the rapid urease test was submitted to DNA extraction with the employment of the GFx DNA extraction kit purchased from Amersham/Pharmacia Biotech, following the manufacturer’s instructions. DNA was quantified in agarose gel electrophoresis using the Invitrogen, Grand Island, New York, USA, low mass ladder and 50-100ug of total DNA were used in the PCR reactions with the oligonucleotides: Hp23Sr6 sense (5′ CACACAGGTAGATGAGATGAGTA3′) and Hp23Sr7 antisense (CACACAGAACCACCGGATCACTA3′), which amplified a fragment of 768pb corresponding to the domain V of the *H. pylori* 23S rDNA (Figure 1). To overcome the problems of extensive genetic polymorphism for precise PCR detection of *H.pylori*, the oligonucleotide construction was performed after a comparative analysis of the 23S rDNA from *H.pylori* and related organisms available at Genebank on MegAlign Lasergene software. PCR condition was 94°C 5′ followed by 40 cycles of 94°C 30″/60°C 30″/72°C 30″ and one cycle at 72°C 7′, with a total volume of 25 μl containing 1× PCR buffer, 200 μM dNTPs, 2,0 mM MgCl_2_, 1 μM of each oligonucleotide, 1,25 U Taq DNA Polimerase Platinum Brazil (Invitrogen). In all PCR reactions a negative and a positive control were used corresponding to, respectively, sterile water and *H. pylori* PCR positive gastric biopsies. The amplified fragments were digested with M*boII* and B*saI* (New England Biolabs). These enzymes distinguish mutations in the *H. pylori* domain V of the 23S rDNA at the positions 2142 and 2143, respectively. In the presence of A2142G mutation the resulting restriction DNA fragments are of 418 bp and 350 bp and in the presence of A2143G mutation the resulting fragments are of 108, 310 e 350 bp. As a control of M*boII* digestion we used a PCR amplified DNA fragment of 601 bp corresponding to the *Leishmania major* chitinase gene that contains a restriction site for M*boII*. A conserved B*saI* restriction site at the 768 bp PCR amplicon is the positive control for digestion with this enzyme producing DNA fragments of 108 and 660 bp in the absence of A2143G mutation. The products of PCR reactions and restriction analysis were resolved in 1,5% agarose gels, stained with ethidium bromide and photographed under UV light. 23S rDNA 768 bp PCR amplicons from four gastric biopsies (two positive and two negative for *H. pylori* histologic test) with clarithromycin sensitive M*boII* and B*saI* restriction pattern, and from ten gastric biopsies with clarytromycin resistant M*boII* (three samples) and B*saI* (seven samples) restriction patterns were submitted to sequencing with DyeTM Terminator v3.0 cycle Sequencing Ready Reaction kit and an ABI-3100 machine purchased from Applied Biosystem, according to the manufacturer’s instructions. Nucleotide sequence determination was performed in duplicate and comparative analysis was carried out by basic nucleotide BLAST alignment [42].

### Statistical analysis

*H. pylori* diagnostic tests were evaluated by calculating sensitivity, specificity and accuracy employing histology as the gold standard.

## Results and discussion

This is the first Brazilian large scale study on *H. pylori* diagnosis and clarithromycin resistance directly from biopsy specimens of 1137 consecutive patients submitted to upper gastroscopy, over a four year period, in a city in the interior of São Paulo, Brazil.

Gastric disease outcome of all patients enrolled in this study attended at the gastroenterology outpatient clinic of Hospital das Clínicas of Marília was investigated by endoscopy and histopathology. Endoscopic finds of peptic or duodenal ulcer disease (PUD) was present in 123 patients. Different degrees of chronic gastritis (CG) were observed by histopathology in 706 patients and normal gastric mucosa, associated or not to gastroesophageal reflux disease (GERD) was found in 290 patients. Eighteen patients were diagnosed as having adenocarcinoma (15) and MALT lymphoma (3) and were excluded from the study. Epidemiological analysis, clinical outcome and *H. pylori* prevalence of these samples were recently published [43].

Detection of *H. pylori* was performed directly from biopsy specimens by three different tests: histology, a household rapid urease test and PCR with the primers Hp23Sr6/r7 which amplify a 768 bp bacterium fragment of the domain V of the 23S rDNA. Histology is the gold standard *H.pylori* diagnostic test employed in our clinical routine which together with histopathological analysis is used to decide for *H. pylori* eradication therapy. The household rapid urease test showed a very low positive predictive value for *H. pylori* associated gastric diseases and a high discrepancy when compared to histology; consequently these data were excluded from the study (data not shown). The 23S rDNA PCR method detected *H. pylori* in 488 gastric biopsies specimens where histology was positive for 451 biopsies samples. Comparative analysis of the PCR assay performed with the Hp23Sr6/r7 with histology showed sensitivity, specificity and accuracy of 77,6%, 79,3% and 78,6%, respectively (Table 1). As both tests were performed on a single and different biopsy and *H. pylori* infection presents a focal characteristic of infection [44,45], accuracy of 78,6% is acceptable for a trustworthy diagnostic test. It can be demonstrated by consistence of the *H.pylori* detection by PCR and histology employed in CG (53,1% and 52,7%, respectively) and PUD (61,2% and 62,6%, respectively) patients (Table 1). PCR detected *H.pylori* in 12,75% in patients with normal gastric mucosa while histology was positive for only 0,8% of the samples. These results can be explained by the more sensitive characteristic of the acid nucleic based method. In order to improve the diagnosis of *H.pylori* some authors suggest the analysis of multiple biopsies [44]. In order to confirm the specificity of the PCR fragments obtained, amplicons from two samples with histologic test for *H. pylori* positive and two samples with histologic test for *H. pylori* negative were sequenced. BLASTN analysis of all four biopsy amplified PCR fragments with the 23S rDNA *H. pylori* specific primers [Genbank:KF680642, Genbank:KF680643, Genbank:KF680644 and Genbank:KF680645] revealed identity of 100% with the 23S rDNA referent to different strains of *H. pylori*. Accordingly, the developed PCR assay is rapid and accurate and can be used as a practical method for the detection of *H.pylori* infection.

**Table 1:** **Clinical outcome and comparison of *H. pylori *** **diagnostic methods**

	** PUD (n = 123)**			** CG (n = 706)**			** N (n = 290)**			
	**His+**	**His-**		**His+**	**His-**		**His+**	**His-**		**T**
**PCR+**	63	13	76	286	89	375	1	36	37	488
**PCR-**	14	33	47	86	245	331	1	252	253	631
**T**	77	46	123	372	334	706	2	288	290	1119

*CG* chronic gastritis, *PUD* peptic ulcer disease, *N* normal gastric mucosa associated or not to gastroesophageal reflux disease (GERD), *PCR* polymerase chain reaction, *His* histology.

Antibiotic treatment of gastric diseases is recommended when *H. pylori* diagnosis is positive and the bacterium classic eradication therapy composed of clarithromycin, amoxicillin and a pump proton inhibitor is prescribed. The chosen therapy present a high failure of *H.pylori* eradication rate in areas where resistance to clarithromycin is higher than 15%, probably in response to the widespread use of this antibiotic for respiratory tract infection, especially in children [9]. Global primary resistance of *H. pylori* to clarithromycin ranges from 1 to 29% [46]. In Brazil, several studies reported a clarithromycin resistance prevalence of 7-16% in adults [19,20,22,47] and 27% in children [23]. Thus, in order to improve the empirical choice of *H.pylori* associated disease therapy, we investigated the regional rate of *H.pylori* clarithromycin resistance through detection of the major related point mutations, A2142G and A2143G at domain V of the *H. pylori* 23S rDNA.

Thus, all 488 *H.pylori* PCR positive samples were analyzed by RFLP of the 768 bp PCR fragment obtained with primers Hp23Sr6/7 with the restriction enzymes M*boII* and B*saI*, with detect mutations A2142G and A2143G at domain V of the *H. pylori* 23S rDNA, respectively (Figure 1). Only 12 samples (2,46%) showed the mutated restriction pattern, three (25%) harboring A2142G mutation, seven (58,3%) A2143G and one sample (8,7%) showed both rDNA point mutations in the PCR 23S rDNA 768 bp fragment. One sample showed partial digestion with the enzyme M*boII* (Figure 1). The point mutations A2142G and A2143G of the amplicons obtained from three [Genbank:KF680646, Genbank:KF680647 and Genbank:KF680647] and seven [Genbank:680649, Genbank:680650, Genbank:680651, Genbank:680652, Genbank:680653, Genbank:680654 and Genbank:680655] different biopsies samples, respectively, were confirmed by sequencing. There was no association of clarithromycin *H.pylori* resistance point mutations with patients’ age or gender (data not shown).

The prevalence of *H. pylori* clarithromycin resistance found in our region was similar to that found in developed countries such as Italy and Germany [7] and in the South American developing country Paraguay [48]. These results confirm the high regional variability of *H.pylori* antibiotic resistance and spite of increasing clarithromycin resistance worldwide, in Marilia, a low resistance rate was maintained over the period of four years. Moreover, PCR/RFLP was a rapid and accurate method for the detection of clarithromycin resistance through a gene mutation directly in gastric biospsy samples and can be used together with histology to decide for prescription of clarithromycin containing regimen therapy.

*H. pylori* 23S rRNA domain V A2142G and A2143G point mutations are the major mutations found in *H. pylori* clinical isolates resistant to clarithromycin. We found a higher prevalence of A2143G compared to A2142G mutation in our samples which is in agreement with the majority of Brazilian studies including the States of Minas Gerais, São Paulo and Recife [20,34,36]. A2143G but not A2142G point mutation shows a synergistic effect of clarithromycin and amoxicillin, which have been used together in the first-line *H. pylori* regimen [49], reinforcing the necessity to investigate this clarithromycin 23S rDNA point mutation before treatment. One sample harbored both A2142G and A2143G mutations at domain V of the *H. pylori* 23S rDNA that was also found in three *H.pylori* isolates obtained from patients of the Brazilian State of Minas Gerais [20]. These results together with the occurrence of partial digestion of a 768 bp 23S rDNA PCR fragment (Figure 1) can be indicative of stomach colonization of multiple strains of *H.pylori*[50].

In Minas Gerais, Brazil, clarithromycin resistance has increased from 4,48% in 1996 to 19,05% in 2000 [20], probably due to the use of macrolides in the treatment of other infectious diseases. We did not find any significant difference in samples resistant to clarithromycin according to the period of the study (data not shown). These data indicate that in our region the prescription and utilization of macrolides is not performed on a high scale. More studies are necessary to confirm this hypothesis.

Clarithromycin resistance reduces the clinical efficacy of clarithromycin-based triple therapy. However, as prevalence of primary resistance of *H.pylori* to clarithromycin due to the rDNA 23S A2142G and A2143G nucleotide substitutions remains low in Marilia, the standard clarithromycin containing triple therapy is still valid as the most effective empirical first-line eradication therapy for *H. pylori* infection.

## Conclusions

The developed PCR assay targeted to the 23S rDNA gene of *H. pylori* is rapid and accurate and can be used as a practical method for the detection of *H.pylori* infection directly on gastric biopsy specimens. Furthermore, the *H. pylori* 23S rDNA PCR fragment obtained can be used to detect point mutations from 1728 to 2495 bp of the *H. pylori* 23S rDNA domain V associated to clarithromycin resistance. Prevalence of primary resistance of *H.pylori* to clarithromycin due to 23S rDNA A2142G and A2143G nucleotide substitutions remains low in Marilia, thus the standard clarithromycin containing triple therapy is still valid as the most effective empirical first-line eradication therapy for *H. pylori* infection.

## Abbreviations

PUD: Peptic ulcer disease; CG: Chronic gastritis; GERD: Gastroesophageal reflux disease; PCR: Polymerase chain reaction; RFLP: Restriction fragment length polymorphism.

## Competing interests

The authors declare that they have no competing interest.

## Authors’ contributions

RBS carried out the processing of the samples, molecular studies, interpretation of data and participated to the draft of the manuscript. RABL, GACL and THH carried out the molecular studies and contributed to the acquisition and interpretation of molecular data; MAS designed the experiments, contributed to data analysis and drafted the manuscript. All authors read and approved the final manuscript.

## Pre-publication history

The pre-publication history for this paper can be accessed here:

http://www.biomedcentral.com/1471-230X/13/164/prepub

## References

[B1] Megraud F Helicobacter pylori infection: Review and practice Presse Med 2010 39 7–8 815 822 20627443 10.1016/j.lpm.2010.04.004

[B2] Wilhelmsen I Berstad A Quality of life and relapse of duodenal ulcer before and after eradication of Helicobacter pylori Scand J Gastroenterol 1994 29 10 874 879 10.3109/00365529409094856 7839092

[B3] Na HS Hong SJ Yoon HJ Maeng JH Ko BM Jung IS Ryu CB Kim JO Cho JY Lee JS Eradication rate of first-line and second-line therapy for Helicobacter pylori infection, and reinfection rate after successful eradication Korean J Gastroenterol 2007 50 3 170 175 17885282

[B4] Chisholm SA Teare EL Davies K Owen RJ Surveillance of primary antibiotic resistance of Helicobacter pylori at centres in England and Wales over a six-year period (2000–2005) Euro Surveill 2007 12 7 E3 4 17991408 10.2807/esm.12.07.00721-en

[B5] Coelho LG Zaterka S Second Brazilian Consensus Conference on Helicobacter pylori infection Arq Gastroenterol 2005 42 2 128 132 16127570 10.1590/s0004-28032005000200012

[B6] McNamara D O’Morain C Consensus guidelines: agreement and debate surrounding the optimal management of Helicobacter pylori infection Can J Gastroenterol 2000 14 6 511 517 10888732 10.1155/2000/604563

[B7] Malfertheiner P Megraud F O’Morain C Bazzoli F El-Omar E Graham D Hunt R Rokkas T Vakil N Kuipers EJ Current concepts in the management of Helicobacter pylori infection: the Maastricht III Consensus Report Gut 2007 56 6 772 781 10.1136/gut.2006.101634 17170018 PMC1954853

[B8] Chey WD Wong BC American College of Gastroenterology guideline on the management of Helicobacter pylori infection Am J Gastroenterol 2007 102 8 1808 1825 10.1111/j.1572-0241.2007.01393.x 17608775

[B9] Rimbara E Fischbach LA Graham DY Optimal therapy for Helicobacter pylori infections Nat Rev Gastroenterol Hepatol 2011 8 2 79 88 10.1038/nrgastro.2010.210 21293508

[B10] Graham DY Fischbach L Helicobacter pylori infection N Engl J Med 2010 363 6 595 596 author reply 596 20836243 10.1056/NEJMc1006158

[B11] Qasim A O’Morain CA Review article: treatment of Helicobacter pylori infection and factors influencing eradication Aliment Pharmacol Ther 2002 16 Suppl 1 24 30 11849124 10.1046/j.1365-2036.2002.0160s1024.x

[B12] Wermeille J Cunningham M Dederding JP Girard L Baumann R Zelger G Buri P Metry JM Sitavanc R Gallaz L Failure of Helicobacter pylori eradication: is poor compliance the main cause? Gastroenterol Clin Biol 2002 26 3 216 219 11981460

[B13] Egan BJ Marzio L O’Connor H O’Morain C Treatment of Helicobacter pylori infection Helicobacter 2008 13 Suppl 1 35 40 18783520 10.1111/j.1523-5378.2008.00639.x

[B14] Silva FM Eisig JN Teixeira AC Barbuti RC Navarro-Rodriguez T Mattar R Short-term triple therapy with azithromycin for Helicobacter pylori eradication: low cost, high compliance, but low efficacy BMC Gastroenterol 2008 8 20 10.1186/1471-230X-8-20 18510773 PMC2438368

[B15] Broutet N Tchamgoue S Pereira E Lamouliatte H Salamon R Megraud F Risk factors for failure of Helicobacter pylori therapy–results of an individual data analysis of 2751 patients Aliment Pharmacol Ther 2003 17 1 99 109 10.1046/j.1365-2036.2003.01396.x 12492738

[B16] Kato M Yamaoka Y Kim JJ Reddy R Asaka M Kashima K Osato MS El-Zaatari FA Graham DY Kwon DH Regional differences in metronidazole resistance and increasing clarithromycin resistance among Helicobacter pylori isolates from Japan Antimicrob Agents Chemother 2000 44 8 2214 2216 10.1128/AAC.44.8.2214-2216.2000 10898707 PMC90045

[B17] Lee JH Shin JH Roe IH Sohn SG Kang GH Lee HK Jeong BC Lee SH Impact of clarithromycin resistance on eradication of Helicobacter pylori in infected adults Antimicrob Agents Chemother 2005 49 4 1600 1603 10.1128/AAC.49.4.1600-1603.2005 15793150 PMC1068646

[B18] Giorgio F Principi M De Francesco V Zullo A Losurdo G Di Leo A Ierardi E Primary clarithromycin resistance to Helicobacter pylori: Is this the main reason for triple therapy failure? World journal of gastrointestinal pathophysiology 2013 4 3 43 46 10.4291/wjgp.v4.i3.43 23946886 PMC3740258

[B19] Mendonca S Ecclissato C Sartori MS Godoy AP Guerzoni RA Degger M Pedrazzoli J Jr Prevalence of Helicobacter pylori resistance to metronidazole, clarithromycin, amoxicillin, tetracycline, and furazolidone in Brazil Helicobacter 2000 5 2 79 83 10.1046/j.1523-5378.2000.00011.x 10849055

[B20] Prazeres Magalhaes P De Magalhaes Queiroz DM Campos Barbosa DV Aguiar Rocha G Nogueira Mendes E Santos A Valle Correa PR Camargos Rocha AM Martins Teixeira L Affonso de Oliveira C: Helicobacter pylori primary resistance to metronidazole and clarithromycin in Brazil Antimicrob Agents Chemother 2002 46 6 2021 2023 10.1128/AAC.46.6.2021-2023.2002 12019131 PMC127243

[B21] Ribeiro ML Vitiello L Miranda MC Benvengo YH Godoy AP Mendonca S Pedrazzoli J Jr Mutations in the 23S rRNA gene are associated with clarithromycin resistance in Helicobacter pylori isolates in Brazil Ann Clin Microbiol Antimicrob 2003 2 11 10.1186/1476-0711-2-11 14633281 PMC305369

[B22] Eisig JN Silva FM Barbuti RC Navarro-Rodriguez T Moraes-Filho JP Pedrazzoli J Jr Helicobacter pylori antibiotic resistance in Brazil: clarithromycin is still a good option Arq Gastroenterol 2011 48 4 261 264 10.1590/S0004-28032011000400008 22147131

[B23] Garcia GT Aranda KR Goncalves ME Cardoso SR Iriya K Silva NP Scaletsky IC High prevalence of clarithromycin resistance and cagA, vacA, iceA2, and babA2 genotypes of Helicobacter pylori in Brazilian children J Clin Microbiol 2010 48 11 4266 4268 10.1128/JCM.01034-10 20826649 PMC3020848

[B24] Megraud F Current recommendations for Helicobacter pylori therapies in a world of evolving resistance Gut Microbes 2013 4 6 10.4161/gmic.25930 PMC3928164 23929066

[B25] Woo HY Park DI Park H Kim MK Kim DH Kim IS Kim YJ Dual-priming oligonucleotide-based multiplex PCR for the detection of Helicobacter pylori and determination of clarithromycin resistance with gastric biopsy specimens Helicobacter 2009 14 1 22 28 10.1111/j.1523-5378.2009.00654.x 19191892

[B26] Chisholm SA Owen RJ Teare EL Saverymuttu S PCR-based diagnosis of Helicobacter pylori infection and real-time determination of clarithromycin resistance directly from human gastric biopsy samples J Clin Microbiol 2001 39 4 1217 1220 10.1128/JCM.39.4.1217-1220.2001 11283030 PMC87913

[B27] Tajbakhsh S Samarbaf-Zadeh AR Moosavian M Comparison of fluorescent in situ hybridization and histological method for the diagnosis of Helicobacter pylori in gastric biopsy samples Med Sci Monit 2008 14 9 BR183 187 18758410

[B28] Burucoa C Garnier M Silvain C Fauchere JL Quadruplex real-time PCR assay using allele-specific scorpion primers for detection of mutations conferring clarithromycin resistance to Helicobacter pylori J Clin Microbiol 2008 46 7 2320 2326 10.1128/JCM.02352-07 18463216 PMC2446943

[B29] Suzuki RB Almeida CM Speranca MA Absence of Helicobacter pylori high tetracycline resistant 16S rDNA AGA926-928TTC genotype in gastric biopsy specimens from dyspeptic patients of a city in the interior of Sao Paulo, Brazil BMC Gastroenterol 2012 12 49 10.1186/1471-230X-12-49 22594560 PMC3449196

[B30] Moazed D Noller HF Chloramphenicol, erythromycin, carbomycin and vernamycin B protect overlapping sites in the peptidyl transferase region of 23S ribosomal RNA Biochimie 1987 69 8 879 884 10.1016/0300-9084(87)90215-X 3122849

[B31] Taylor DE Ge Z Purych D Lo T Hiratsuka K Cloning and sequence analysis of two copies of a 23S rRNA gene from Helicobacter pylori and association of clarithromycin resistance with 23S rRNA mutations Antimicrob Agents Chemother 1997 41 12 2621 2628 9420030 10.1128/aac.41.12.2621 PMC164180

[B32] Versalovic J Shortridge D Kibler K Griffy MV Beyer J Flamm RK Tanaka SK Graham DY Go MF Mutations in 23S rRNA are associated with clarithromycin resistance in Helicobacter pylori Antimicrob Agents Chemother 1996 40 2 477 480 8834903 10.1128/aac.40.2.477 PMC163139

[B33] Stone GG Shortridge D Flamm RK Versalovic J Beyer J Idler K Zulawinski L Tanaka SK Identification of a 23S rRNA gene mutation in clarithromycin-resistant Helicobacter pylori Helicobacter 1996 1 4 227 228 10.1111/j.1523-5378.1996.tb00043.x 9432314

[B34] Toracchio S Aceto GM Mariani-Costantini R Battista P Marzio L Identification of a novel mutation affecting domain V of the 23S rRNA gene in Helicobacter pylori Helicobacter 2004 9 5 396 399 10.1111/j.1083-4389.2004.00267.x 15361077

[B35] Fontana C Favaro M Minelli S Criscuolo AA Pietroiusti A Galante A Favalli C New site of modification of 23S rRNA associated with clarithromycin resistance of Helicobacter pylori clinical isolates Antimicrob Agents Chemother 2002 46 12 3765 3769 10.1128/AAC.46.12.3765-3769.2002 12435674 PMC132752

[B36] Rimbara E Noguchi N Kawai T Sasatsu M Novel mutation in 23S rRNA that confers low-level resistance to clarithromycin in Helicobacter pylori Antimicrob Agents Chemother 2008 52 9 3465 3466 10.1128/AAC.00445-08 18606842 PMC2533477

[B37] Scaletsky IC Aranda KR Garcia GT Goncalves ME Cardoso SR Iriya K Silva NP Application of real-time PCR stool assay for Helicobacter pylori detection and clarithromycin susceptibility testing in Brazilian children Helicobacter 2011 16 4 311 315 10.1111/j.1523-5378.2011.00845.x 21762271

[B38] Lins AK Lima RA Magalhaes M Clarithromycin-resistant Helicobacter pylori in Recife, Brazil, directly identified from gastric biopsies by polymerase chain reaction Arquivos de gastroenterologia 2010 47 4 379 382 10.1590/S0004-28032010000400011 21225149

[B39] van Doorn LJ Glupczynski Y Kusters JG Megraud F Midolo P Maggi-Solca N Queiroz DM Nouhan N Stet E Quint WG Accurate prediction of macrolide resistance in Helicobacter pylori by a PCR line probe assay for detection of mutations in the 23S rRNA gene: multicenter validation study Antimicrob Agents Chemother 2001 45 5 1500 1504 10.1128/AAC.45.5.1500-1504.2001 11302817 PMC90495

[B40] Assumpcao MB Martins LC Melo Barbosa HP Barile KA de Almeida SS Assumpcao PP Corvelo TC Helicobacter pylori in dental plaque and stomach of patients from Northern Brazil World J Gastroenterol 2010 16 24 3033 3039 10.3748/wjg.v16.i24.3033 20572307 PMC2890944

[B41] Rotimi O Cairns A Gray S Moayyedi P Dixon MF Histological identification of Helicobacter pylori: comparison of staining methods J Clin Pathol 2000 53 10 756 759 10.1136/jcp.53.10.756 11064668 PMC1731087

[B42] Altschul SF Madden TL Schaffer AA Zhang J Zhang Z Miller W Lipman DJ Gapped BLAST and PSI-BLAST: a new generation of protein database search programs Nucleic Acids Res 1997 25 17 3389 3402 10.1093/nar/25.17.3389 9254694 PMC146917

[B43] Suzuki RB Cola RF Cola LT Ferrari CG Ellinger F Therezo AL Silva LC Eterovic A Speranca MA Different risk factors influence peptic ulcer disease development in a Brazilian population World J Gastroenterol 2012 18 38 5404 5411 10.3748/wjg.v18.i38.5404 23082057 PMC3471109

[B44] Morris A Ali MR Brown P Lane M Patton K Campylobacter pylori infection in biopsy specimens of gastric antrum: laboratory diagnosis and estimation of sampling error J Clin Pathol 1989 42 7 727 732 10.1136/jcp.42.7.727 2474579 PMC1142023

[B45] Sugiyama T Sakaki N Kozawa H Sato R Fujioka T Satoh K Sugano K Sekine H Takagi A Ajioka Y Sensitivity of biopsy site in evaluating regression of gastric atrophy after Helicobacter pylori eradication treatment Aliment Pharmacol Ther 2002 16 Suppl 2 187 190 11966540 10.1046/j.1365-2036.16.s2.17.x

[B46] Megraud F Lehours P Helicobacter pylori detection and antimicrobial susceptibility testing Clin Microbiol Rev 2007 20 2 280 322 10.1128/CMR.00033-06 17428887 PMC1865594

[B47] Godoy AP Ribeiro ML Benvengo YH Vitiello L Miranda Mde C Mendonca S Pedrazzoli J Jr Analysis of antimicrobial susceptibility and virulence factors in Helicobacter pylori clinical isolates BMC Gastroenterol 2003 3 20 10.1186/1471-230X-3-20 12911839 PMC194586

[B48] Farina N Kasamatsu E Samudio M Moran M Sanabria R Laspina F Antimicrobial susceptibility of H pylori strains obtained from Paraguayan patients Revista medica de Chile 2007 135 8 1009 1014 17989858 10.4067/s0034-98872007000800008

[B49] Sakinc T Baars B Wuppenhorst N Kist M Huebner J Opferkuch W Influence of a 23S ribosomal RNA mutation in Helicobacter pylori strains on the in vitro synergistic effect of clarithromycin and amoxicillin BMC research notes 2012 5 603 10.1186/1756-0500-5-603 23110798 PMC3522010

[B50] Figueiredo C Van Doorn LJ Nogueira C Soares JM Pinho C Figueira P Quint WG Carneiro F Helicobacter pylori genotypes are associated with clinical outcome in Portuguese patients and show a high prevalence of infections with multiple strains Scand J Gastroenterol 2001 36 2 128 135 11252403 10.1080/003655201750065861

